# Rates of switching to second-line antiretroviral therapy and impact of delayed switching on immunologic, virologic, and mortality outcomes among HIV-infected adults with virologic failure in Rakai, Uganda

**DOI:** 10.1186/s12879-017-2680-6

**Published:** 2017-08-22

**Authors:** Victor Ssempijja, Gertrude Nakigozi, Larry Chang, Ron Gray, Maria Wawer, Anthony Ndyanabo, Jingo Kasule, David Serwadda, Barbara Castelnuovo, Anja van’t Hoog, Steven James Reynolds

**Affiliations:** 1 Clinical Research Directorate/Clinical Monitoring Research Program, Leidos Biomedical Research, Inc., NCI Campus at Frederick, Frederick, MD 21702 USA; 2 Rakai Health Sciences Program, Kalisizo, Uganda; 3 Johns Hopkins School of Medicine, Baltimore, MD USA; 4 Johns Hopkins Bloomberg School of Public Health, Baltimore, MD USA; 5 School of Public Health, Makerere University College of Health Sciences, Kampala, Uganda; 6 Infectious Disease Institute, Makerere University College of Health Sciences, Kampala, Uganda; 7 Department of Global Health –Academic Medical Center, University of Amsterdam, Amsterdam, Netherlands; 8 Division of Intramural Research, National Institute of Allergy and Infectious Diseases, National Institutes of Health, Bethesda, MD USA; 9 Clinical Monitoring Research Program (CMRP), Leidos Biomedical Research, Inc., 5705 Industry Lane, Frederick, MD 21704 USA

**Keywords:** Antiretroviral therapy, Virologic failure, Treatment switch, HIV, Cohort studies, Competing risk model, Mortality, Second line antiretroviral therapy

## Abstract

**Background:**

Switch from first to second-line ART is recommended by WHO for patients with virologic failure. Delays in switching may contribute to accumulated drug resistance, advanced immunosuppression, increased morbidity and mortality. The 3rd 90′ of UNAIDS 90:90:90 targets 90% viral suppression for persons on ART. We evaluated the rate of switching to second-line antiretroviral therapy (ART), and the impact of delayed switching on immunologic, virologic, and mortality outcomes in the Rakai Health Sciences Program (RHSP) Clinical Cohort Study which started providing ART in 2004 and implemented 6 monthly routine virologic monitoring beginning in 2005.

**Methods:**

Retrospective cohort study of HIV-infected adults on first-line ART who had two consecutive viral loads (VLs) >1000 copies/ml after 6 months on ART between June 2004 and June 2011 was studied for switching to second-line ART. Immunologic decline after virologic failure was defined as decrease in CD4 count of ≥50 cells/ul and virologic increase was defined as increase of 0.5 log 10 copies/ml. Competing risk models were used to summarize rates of switching to second-line ART while cox proportional hazard marginal structural models were used to assess the risk of virologic increase or immunologic decline associated with delay to switch first line ART failing patients.

**Results:**

The cumulative incidence of switching at 6, 12, and 24 months following virologic failure were 30.2%, 44.6%, and 65.0%, respectively. The switching rate was increased with higher VL at the time of virologic failure; compared to those with VLs ≤ 5000 copies/ml, patients with VLs = 5001–10,000 copies/ml had an aHR = 1.81 (95% CI = 0.9–3.6), and patients with VLs > 10,000 copies/ml had an aHR = 3.38 (95%CI = 1.9–6.2). The switching rate was also increased with CD4 < 100 cells/ul at ART initiation, compared to those with CD4 ≥ 100 cells/ul (aHR = 2.30, 95% CI = 1.5–3.6). Mortality in patients not switched to second-line ART was 11.9%, compared to 1.2% for those who switched (*p* = 0.009). Patients switched after 12 months of of virologic failure were more likely to experience CD4 decline and/or further VL increases.

**Conclusions:**

Intervention strategies that aid clinicians to promptly switch patients to second-line ART as soon as virologic failure on 1st line ART is confirmed should be prioritized.

**Electronic supplementary material:**

The online version of this article (doi:10.1186/s12879-017-2680-6) contains supplementary material, which is available to authorized users.

## Background

The World Health Organization (WHO) recommends switching from first- to second-line antiretroviral therapy (ART) for HIV patients with virologic failure [1] to avert drug resistance, advanced immunosuppression, increased morbidity and mortality, and to reduce the risk of transmitting HIV to uninfected sex partners [2–7]. In order to promptly switch treatment and to ensure sustained virologic suppression, viral load (VL) monitoring to identify virologic failure is crucial. WHO defines virologic failure as two consecutive VLs > 1000 copies/ml after 6 or more months on ART [1, 8].

Analyses of rates and determinants of switching to second-line ART have largely been estimated for the entire patient population on ART rather than restricted to patients with proven virologic failure. Studies for overall ART populations reported switching rates of 2.6–3.3/100 person years (pys) [9, 10]. Factors associated with switching to second-line ART include drug intolerance, virologic failure, and rapid decline in CD4 count after ART initiation [9, 11]. A pooled analysis of switching to second-line ART among patients with declining CD4 levels and/or elevated VLs found increased mortality in patients who did not switch [12]. A pooled analysis from several clinics in sub-Saharan Africa found that 58% of patients with confirmed virologic failure were switched within 2 years [11]. However, data on patient-centered outcomes in ART programs with routine virologic monitoring in developing countries are still limited.

Using data from the Rakai Health Sciences Program (RHSP) Clinical Cohort Study, which monitors HIV care programs, we retrospectively assessed the rate and factors associated with switching from first- to second-line ART among HIV-infected adults with virologic failure 6 months after ART initiation, and evaluated the impact of delayed switching on immunologic, virologic, and mortality outcomes.

## Methods

### Analysis design and setting

We conducted a retrospective study of HIV-infected adults initiated on ART between June 2004 and June 2011 in the RHSP clinical cohort, in south-central Uganda. Since 2004, RHSP, with funding from the President’s Emergency Plan for AIDS Relief (PEPFAR), has provided free ART using a community-based decentralized service-delivery model. First-line ART regimens consisted of two nucleoside/nucleotide reverse transcriptase inhibitors (NRTIs; zidovudine or stavudine and lamivudine) and one non-nucleoside reverse transcriptase inhibitor (NNRTI; nevirapine or efavirenz), while second-line consisted of ritonavir-boosted lopinavir with 2 NRTIs. After ART initiation, participants were seen weekly for the first month, then biweekly for 2 months and monthly thereafter, with adherence and HIV risk reduction promotion at all visits, and CD4 and VL monitoring every 6 months. In 2005, RHSP initiated bi-annual routine VL monitoring to identify patients experiencing virologic failure. Interventions to address virologic failure included intensified adherence counseling, peer support, and switching to second-line ART [13]. Decisions about and timing of the switch to second-line ART were made on a case-by-case basis at the clinician’s discretion, and were based on the suspected cause of virologic failure (poor adherence or suspected drug resistance).

### Inclusion criteria, outcomes, and definitions

We included all HIV-infected adults aged 18 years or older who had been on ART for at least 6 months. Operational definitions of virologic failure changed over time so we defined virologic failure based on the current WHO guidelines [8] as two consecutive VLs > 1000 copies/ml detected within 12 months while on ART. Viral suppression was defined as having a VL < 400 copies/ml.

The primary analysis explored the risk factors of switching to second-line ART among patients confirmed to have VF. The primary outcome was a switch to second-line ART after first occurrence of VF defined as the change from an NNRTI-based regimen to a ritonavir-boosted, lopinavir-based regimen, and the primary exposure was time from VF to switching to second-line ART. The secondary analysis focused on patients that switched to second-line ART during the study to evaluate the risk of immunologic decline, virologic increase or death associated with delay in switching to second line ART. We defined an event of immunologic decline as decrease in CD4 count up 50 cells/ul from the CD4 at the time of virologic failure, and event of virologic increase as an increase of viral load count ≥0.5 log10 copies/ml from the VL at the time of virologic failure. We considered deaths that occurred in patients switched to second-line ART. In this analysis, the exposure of interest was time from VF to immunologic decline or virologic increase or death, compared among patients switched 0–6 months, 7–12 months, 13–24 months, ≥25 months.. Other exposure variables of interest included characteristics at ART initiation; age in years (18–24, 25–34, 35+), gender, type of ART treatment clinic defined as *central* if care was received at the large, fixed-location referral clinic/laboratory at the RHSP main facility in Kalisizo, or *peripheral* if care was received at one of 16 HIV treatment clinics conducted by visiting RHSP staff once every two weeks at local government health center facilities, year of ART initiation categorized as 2004–2007 or 2008–2011 to indicate scale-up and stabilized implementation phases of the ART program, WHO stage at ART initiation (I, II, III/IV), CD4 count at ART initiation (<100 or ≥100 cells/ul); characteristics at time of VF: CD4 count (<100 or ≥100 cells/ul), viral load (≤5000, 5001–10,000 or >10,000 copies/ml), year of VF and status of virologic failure prior to VF.

### Statistical analysis

For the primary analysis, person time of observation was computed from the date that the second consecutive (confirmatory) VL of VF was >1000 copies/ml to the time of switching to second-line ART if switched or censored at the last available clinic visit if lost to follow-up or at time of death or administratively censored on April 01, 2014 if not switched, whichever occurred first. Then we used competing risk models to estimate the cumulative incidence and adjusted hazard ratios (aHRs) with 95% confidence intervals (CIs) of switching to the second-line ART regimen following VF, treating death as a competing risk [14]. In the secondary analysis, person time of observation was computed from the date of the second consecutive (confirmatory) VL of VF was >1000 copies/ml to the time of immunologic decline, virologic increase or death for patients that reached the respective end points, or censored at the last available clinic visit if lost to follow-up or at time of death or administratively censored on April 01, 2014 if they didn’t reach the endpoint, whichever came first. We aimed to achieve a hypothetical randomized trial in which patients were enrolled after confirmed VF and randomly assigned to either switch to second-line ART in 0–6, 7–12 months, 13–24 months, 25+ months or delay switching to longer than two years. Given that the VL profile of a patient influences switching to second-line ART (exposure of interest) and also influences the risk of death, future immunologic and virologic outcomes, there is likelihood of confounding by indication. Therefore we used the cox proportional marginal structured models (MSMs) to estimate the association between the exposure and outcomes. VL was considered a time-varying confounder and used to compute inverse probability weights (IPWs) of time to switching and the IPWs of censoring using pooled logistic regression models with robust standard error estimates to account for repeated measures. We adjusted for VL at each visit, duration on first-line ART until VF, year of VF failure, CD4 at ART initiation and VL at time of VF to compute the weights and thereafter stabilized them. Cox proportional hazard MSM adjusted hazard ratios (aHRs) with 95% confidence intervals were estimated separately for the endpoints of immunologic decline and virologic increase, and for a composite endpoint defined as a CD4 decrease of at least 50 cells/ul compared to CD4 at virologic failure, or a virologic increase of at least 0.5 log 10 copies/ml compared to VL at virologic failure or death. Incidence rates of exposure variable were computed using exact poisson regression models and exposure variables with a wald test *p*-value ≤0.15 in the univariate analysis were included in multivariate analyses. In addition, age and gender were maintained in the multivariate analysis irrespective of their statistical significance to ensure adjustment for any age or gender related confounding. Analysis was performed using STATA 14.0 (STATA, Inc., Texas, USA).

## Results

Of 3036 HIV-infected adults who had initiated ART between June 2004 and June 2011, we identified 124 (4.1%) who met the criteria for virologic failure. A median of 5.6 months (IQR = 5.1–5.6) elapsed between the first and second (confirmatory) failing VL. The median confirmatory failing VL was 13,835 copies/ml (IQR = 4695–67,593) at a median time of 16.7 months (IQR = 11.6–22.7) from ART initiation. At time of ART initiation, 53% of clients were aged 25–34 years, 60% were female, 71% were on niverapine first-line based regimens and 37% has a CD4 count less than 100 cells/ul at ART initiation. While at time of virologic failure 91% has a CD4 count greater than 100 cells/ul, 58% had viral loads of greater than 10,000 copies/ul and 54% had achieved virologic suppression prior to virologic failure (Table 1).

**Table 1:** Demographic and clinical characteristics of HIV infected adults experiencing virologic failure

Characteristics	Overall	Not switched	Switched
	n(%)	n(%)	n(%)
Population	124	42(34%)	82(66%)
Age in years			
18–24	14(11%)	3(7%)	11(13%)
25–34	66(53%)	18(43%)	48(59%)
≥ 35	44(35%)	21(50%)	23(28%)
Gender			
Female	75(60%)	23(55%)	52(63%)
Male	49(40%)	19(45%)	30(37%)
Year of ART Initiation			
2004–2007	78(63%)	26(62%)	52(63%)
2008–2011	46(37%)	16(38%)	30(37%)
Type of ART treatment Clinic			
Central Clinic	23(19%)	7(17%)	16(20%)
Peripheral clinic	101(81%)	35(83%)	66(80%)
WHO Stage			
1	34(27%)	10(24%)	24(29%)
2	50(40%)	17(40%)	33(40%)
3 or 4	40(32%)	15(36%)	25(30%)
First Line ART regimen			
EFV based regimen	36(29%)	14(33%)	22(27%)
NVP based regimen	88(71%)	28(67%)	60(73%)
CD4 count at ART initiation (cells/ul)			
≥ 100	78(63%)	33(79%)	45(55%)
≤ 99	46(37%)	9(21%)	37(45%)
CD4 count at ART failure (cells/ul)^a^			
≥ 100	92(91%)	32(94%)	60(90%)
≤ 99	9(9%)	2(6%)	7(10%)
viral load at ART failure (cells/ul)			
≤ 5000	33(27%)	18(43%)	15(18%)
5001–10,000	19(15%)	6(14%)	13(16%)
> 10,000	72(58%)	18(43%)	54(66%)
Year of virologic failure			
2005–2007	37(30%)	11(26%)	26(32%)
2008–2013	87(70%)	31(74%)	56(68%)
Virologic suppression prior to virologic failure			
No	57(46%)	17(40%)	40(49%)
Yes	67(54%)	25(60%)	42(51%)
Time from ART start to virologic failure			
0–12 months	36(29%)	14(33%)	22(27%)
13–24 months	56(45%)	14(33%)	42(51%)
25–36 months	23(19%)	8(19%)	15(18%)
≥ 37 months	9(7%)	6(14%)	3(4%)
Time from virologic failure to Switch (months)			
0–6 months	N/A	N/A	36(44%)
7–12 months	N/A	N/A	16(20%)
13–24 months	N/A	N/A	19(23%)
≥ 25 months	N/A	N/A	11(13%)

^a^33 patients did not have CD4 at time of confirmed virologic failure; *EFV* Efavirenz, *NVP* Nevirapine

### Rates of switching to second-line ART

A total of 82 (66.1%) patients with virologic failure were switched to second-line ART at a rate of 49/100 pys (95% CI = 39.1–60.4). The median timing of switching to second-line ART was 8.1 months (IQR = 3.7–17.0) after virologic failure detection. The cumulative incidences of switching at 6, 12, and 24 months after virologic failure were 30.2% (95% CI = 22.8–39.3), 44.6% (95% CI = 36.1–54.1), and 65.0% (95% CI = 55.7–74.2), respectively.

**Fig. 1:**
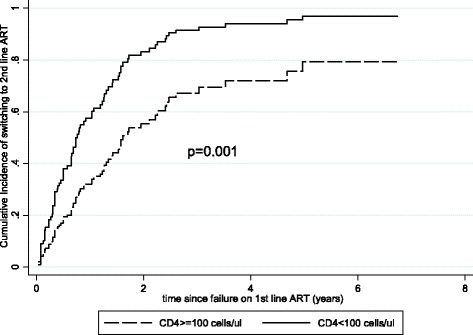
Cumulative incidence of switching to second-line ART by CD4 count at ART initiation

In univariate analyses, significant predictors of more rapid switching to second-line ART were younger age, lower CD4 at ART initiation, and higher VL at the time of virologic failure diagnosis (Table 2). In the adjusted analysis, the adjusted hazard ratio of switching with CD4 < 100 cells/ul at ART initiation relative to patients with ≥100 cells/ul was 2.30, with a 95% CI of 1.5–3.6. Cumulative incidence curves generated from the adjusted competing risk models show a steep increase in the rate of switching in the first 3 years and consistent differences in the rates of switching by the level of CD4 at ART initiation the cumulative incidences of switching at 2 years were ~80% and 40%, respectively (Fig. 1).

**Table 2:** Predictors of Switching to Second-Line ART after Virologic Failure

Characteristics	n/ pys	n/100 pys (95% CI)	Univariate analysis		Multivariate Analysis	
			Hazard ratio (95% CI)	*p*-value	Hazard ratio (95% CI)	*p*-value
Overall	82/168.7	48.6(39.1–60.4)				
Age in years						
18–24	11/12.1	90.9(50.3–164.1)	Ref		Ref	
25–34	48/78.3	61.3(46.2–81.3)	0.85(0.5–1.6)	0.022	0.96(0.5–1.8)	0.254
≥ 35	23/78.3	29.4(19.5–44.2)	0.46(0.2–0.9)		0.63(0.3–1.3)	
Gender						
Female	52/104	50(38.1–65.6)	Ref	0.377	Ref	0.224
Male	30/64.7	46.4(32.4–66.3)	0.82(0.5–1.3)		0.74(0.5–1.2)	
Year of ART initiation						
2004–2007	52/124.4	41.8(31.9–54.9)	Ref	0.239		
2008–2011	30/44.3	67.7(47.3–96.8)	1.31(0.8–2.0)			
Type of ART treatment clinic						
Central clinic	16/21.8	73.5(45–120)	Ref	0.338		
Peripheral clinic	66/146.9	44.9(35.3–57.2)	0.76(0.4–1.3)			
WHO stage at ART initiation						
1	24/45.1	53.2(35.6–79.3)	Ref	0.912		
2	33/75.6	43.7(31–61.4)	0.91(0.6–1.5)			
3 or 4	25/48	52.1(35.2–77.2)	0.90(0.5–1.6)			
First-line ART regimen						
EFV-based regimen	22/43.8	50.2(33.1–76.2)	Ref	0.670		
NVP-based regimen	60/124.9	48(37.3–61.9)	1.11(0.7–1.8)			
CD4 count at ART initiation (cells/ul)						
≥ 100	45/129	34.9(26–46.7)	Ref	<0.001	Ref	<0.001
≤ 99	37/39.7	93.2(67.5–128.6)	2.31(1.5–3.6)		2.30(1.5–3.6)	
CD4 count at ART failure (cells/ul)^a^						
≥ 100	60/141.3	42.5(33–54.7)	Ref	0.316		
≤ 99	7/3.9	180.1(85.8–377.7)	1.57(0.6–3.8)			
Viral load at ART failure (copies/ml)						
≤ 5000	16/76.4	20.9(12.8–34.2)	Ref	<0.001	Ref	<0.001
5001–10,000	12/39	30.8(17.5–54.2)	1.44(0.8–2.7)		1.81(0.9–3.6)	
> 10,000	54/53.3	101.3(77.6–132.2)	3.44(2.0–6.0)		3.38(1.9–6.2)	
Year of virologic failure						
2004–2007	52/124.4	41.8(31.9–54.9)	Ref	0.239		
2008–2013	30/44.3	67.7(47.3–96.8)	1.33(0.8–2.1)			
Virologic suppression prior to virologic failure						
No	52/124.4	41.8(31.9–54.9)	Ref	0.797		
Yes	30/44.3	67.7(47.3–96.8)	0.95(0.6–1.5)			

^a^35 patients did not have CD4 count at time of confirmed virologic failure; *EFV* Efavirenz, *NVP* Nevirapine, n = patients switched to second-line, pys = person years

**Fig. 2:**
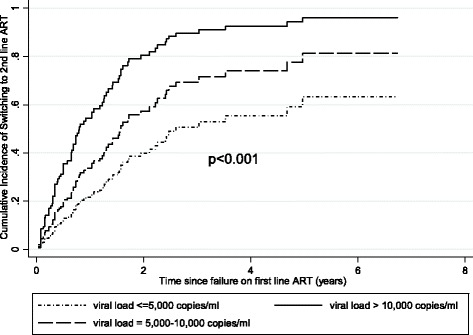
Cumulative incidence of switching to second-line ART by viral load at time of confirmed virologic failure

The rate of switching was also increased with higher VL at the time of virologic failure; compared to patients with VLs ≤ 5000 copies/ml, patients with VLs of 5001–10,000 copies/ml had an aHR of 1.81 and a 95% CI of 0.9–3.6, and patients with VLs > 10,000 copies/ml had an aHR of 3.38 and a 95% CI of 1.9–6.2. Cumulative incidence curves generated from the adjusted competing risk models show a steep increase in the rate of switching in the first three years and consistent differences in the rates of switching by the level of viral load at virologic failure. Corresponding cumulative incidence at 2 years was 40%, 60%, and 80%, respectively (Fig. 2). No other factors were associated with rates of switching (Table 2).

### Mortality associated with switching to second-line ART

Eight deaths occurred among the 124 patients with confirmed virologic failure (6.5%), of which 6 occurred in patients who had not yet switched to second-line ART and 2 were among patients who had been switched to second line ART, translating into mortality rates of 11.9% and 1.22%, respectively (*p* = 0.009).

### Immunologic decline, Virologic increase and death associated with delayed switch to second-line ART

During the follow-up, 30 patients reached the end point of immunologic decline in 259 person years of observation. The overall incidence rate of developing immunologic decline was 11.6/100 pys (95% CI = 8.1–16.6). Patients switched in 0–6 months had an incidence rate of 5.5/100 pys (95% CI = 2.5–12.3), while the incidence rate was 13.6/100 pys (95% CI = 6.5–28.5) among patients switched in 7–12 months, 16.0/100 pys (95% CI = 8.6–29.7) among patients switched in 13–24 months and 19.4/100 pys (95% CI = 9.2–40.7) among patients switched after 24 months since virologic failure. In the adjusted analysis, compared to patients who were switched within 6 months of confirmed virologic failure, patients who switched within 7–12 months and 13–24 months had a non-significantly different hazard ratio of 2.21 (95% CI = 0.4–13.7) and 2.76(95% CI = 0.6–13.7 respectively, while those switched in 25 months or more, experienced significantly higher hazard rate of developing immunologic decline (aHR = 5.11, 95% CI = 1.0–25.2) [Table 3]. Of the candidate confounding variables, age was significantly associated with immunologic decline. Patients aged 18–24 years at ART initiation, those aged 25–34 years had non-significantly different hazards of immunologic decline, while those 35 years or older had a significantly higher hazard rate of immunologic decline (aHRs = 11.14, 95% CI = 1.5–84.2). Other potential confounders found non-significant included; type of ART treatment clinic, WHO stage at ART initiation, CD4 count at ART initiation, first line ART regimen, viral load at first line ART failure, CD4 at first line ART failure, year of virologic failure and virologic suppression prior to virologic failure (Additional files 1, 2 and 3: Tables S1–S3).

**Table 3:** Risk factors associated with delay in switching to second-line ART among HIV infected adults failing on first line ART

Exposure of time to second-line ART	n/ pys	n /100 pys	Univariate analysis		Multivariate Analysis	
		(95% CI)	HRs (95% CI)	*p*-value	HRs (95% CI)	*p*-value
Risk of Immunologic decline associated with time to switch to second-line ART^a^						
Overall	30/259.0	11.6(8.1–16.6)				
Time to 2nd line ART						
0–6 months	6/108.7	5.5(2.5–12.3)	Ref		Ref	
7–12 month	7/51.6	13.6(6.5–28.5)	2.75(0.6–12.1)	0.179	2.21(0.4–13.7)	0.389
13–24 month	10/62.6	16.0(8.6–29.7)	2.51(0.6–10.1)	0.193	2.76(0.6–13.7)	0.212
≥ 25 months	7/36.1	19.4(9.2–40.7)	4.09(1.0–16.9)	0.052	5.11(1.0–25.2)	0.045
Risk of virologic increase associated with time to switch to second-line ART^b^						
Overall	24/270.1	8.9(6.0–13.3)				
Time to 2nd line ART						
0–6 months	4/107.7	3.7(1.4–9.9)	Ref		Ref	
7–12 month	1/75.8	1.3(0.2–9.4)	0.45(0.0–5.3)	0.52	0.32(0.0–3.6)	0.357
13–24 month	9/59.1	15.2(7.9–29.3)	10.40(2.0–52.9)	0.005	10.16(1.9–53.0)	0.006
≥ 25 months	10/27.4	36.5(19.6–67.8)	14.24(2.8–72.2)	0.002	10.13(1.7–59.3)	0.011
Risk of composite end-point associated with time to switch to second-line ART^c^						
Overall	43/212.8	20.2(15.0–27.2)				
Time to 2nd line ART						
0–6 months	10/96.3	10.4(5.6–19.3)	Ref		Ref	
7–12 month	7/51.2	13.7(6.5–28.7)	1.48(0.4–5.8)	0.571	1.06(0.3–3.9)	0.928
13–24 month	15/41.3	36.4(21.9–60.3)	5.27(1.4–19.2)	0.012	5.05(1.5–16.9)	0.009
≥ 25 months	11/24.0	45.8(25.3–82.6)	4.94(1.4–17.1)	0.012	5.58(1.9–16.7)	0.003

Confounders considered for adjusting models a,b and c included: Age, Gender, characteristics at time of ART initiation: type of ART clinic, year of ART initiation, WHO stage (I, II, III/IV.), CD4 count (<100 or ≥100 cells/ul); characteristics at time of VF: CD4 count (<100 or ≥100 cells/ul), viral load (≤5000, 5001–10,000 or >10,000 copies/ml), year of VF and status of virologic failure prior to VF

*EFV* Efavirenz, *NVP* Nevirapine, *n* Number switched to 2nd line, *pys* Person years of observation, *virologic failure* Incident first line ART failure, *HRs* Hazard Ratios

^a^Cox proportional MSM model of the time to event of immunologic decline defined as decrease in CD4 count ≥50 cells/ul above CD4 count at virologic failure

^b^Cox proportional MSM model of the time to event of virologic increase defined as increase in viral load ≥0.5 log 10 copies/ml above viral load at virologic failure

^c^Cox proportional MSM model of the time to event of composite endpoint defined as reaching immunologic decline or virologic increase as defined in a and b above, or dying

When the end point was virologic increase defined as an increase of 0.5 log 10 copies/ml above VL at time of virologic failure, 24 patients reached the end point in 270.1 person years of observation (incidence = 8.9 cases/100 pys, 95% CI = 6.0–13.3) . Patients switched in 0–6 months had an incidence rate of 3.7/100 pys (95% CI = 1.4–9.9), those switched in 7–12 months had an incidence rate of 1.3/100 pys (95% CI = 0.2–9.4) while those switched in 13–24 months and 25 or more months had incidence of 15.2/100 pys (95% CI = 7.9–29.3) and 36.4(95% CI = 19.6–67.8) respectively. In the adjusted analysis, compared to patients who were switched within 6 months of confirmed virologic failure, patients who switched within 7–12 months had a non-significantly different hazard ratio of 0.32 (95% CI = 0.0–3.6), while those switched in 13–34 and 25 months or more experienced significantly higher hazard rate of developing virologic increase (aHR = 10.16, 95% CI = 1.9–53.0) and aHR = 10.13, 95% CI = 1.7–59.3 respectively) [Table 3]. All potential confounders evaluated had non-significant association with incidence of virologic increase (Additional files 1, 2 and 3: Tables S1–S3).

Lastly, we evaluated the risk of the composite endpoint associated with timing to switch to second-line ART. The Two deaths occurred in patients that switched to second line, the overall incidence rate was 20.2/100 pys (95% CI = 15.0–27.2). Patients switched in 0–6 months had an incidence rate of 10.4/100 pys (95% CI = 5.6–19.3), while the incidence rate was 13.7/100 pys (95% CI = 6.5–28.7) among patients switched in 7–12 months, 36.4/100 pys (95% CI = 21.9–60.3) among patients switched in 13–24 months and 45.8/100 pys (95% CI = 25.3–82.6) among patients switched after 24 months since virologic failure. In the adjusted analysis, compared to patients who were switched within 6 months of confirmed virologic failure, patients who switched within 7–12 months had a non-significantly different hazard ratio of 1.06 (95% CI = 0.3–3.9), while those switched with 13–24 months experienced significantly higher hazard rate of dying or developing immunologic or virologic decline (aHR = 5.05, 95% CI = 1.5–16.9), as did those who switched after 24 months (aHR = 5.58, 95% CI = 1.9–16.7) [Table 3]. All potential confounders evaluated had non-significant association with the composite endpoint (Additional files 1, 2 and 3: Tables S1–S3).

## Discussion

We assessed the rate of switching to second-line ART after confirmed virologic failure, identified predictors of regimen change, and explored the impact of delayed switching on immunologic, virologic, and mortality outcomes. We found that switching to second-line ART regimens after virologic failure was delayed beyond 12 months in 55% of patients, which was consistent with reports from other settings [11, 12]. Switching to second-line ART was more rapid in patients with lower CD4 at ART initiation or with higher VLs at virologic failure, and these findings are comparable to other studies [9, 11, 12, 15].

We also found that delay in switching to second-line ART beyond 12 months compared to switching within first six months following virologic failure was associated with more than a 5-fold increased risk of developing either immunologic decline, virologic increase or death. Therefore, delayed switching is not only associated with death as previously documented [3, 12, 16], but also is associated with deteriorating immunologic status of patients which exposes them to opportunistic infections [2], the risk of developing resistance and potential onward transmission to partners.

The goal of effective anti-retroviral therapy is to dramatically reduce the risk of death and morbidity due to HIV, tremendous gains in reaching this goal globally have been achieved. Our results and others [12, 15] show increased mortality when switching to second-line therapy is delayed highlighting an important implementation challenge faced by mature ART treatment programs as they continue to strive towards averting deaths in HIV infected persons on ART.

We found the rate of switching to be 49/100 pys, indicating that up to 51/100 pys are spent on failing first-line regimens after confirmed virologic failure. This finding highlights an important gap that has potential to undercut the gains made in the context of test and start all HIV infected programing that aims to reduce the time of unsuppressed HIV viral load from onset of HIV infection [17]. Therefore to ensure continued virological suppression beyond test and start, it will be necessary to ensure timely switch of regimens when indicated to reduce the time spent on failing ART regimens when the VL is unsuppressed.

There are several factors that could have caused delayed switching in our study. First, as suggested by our findings, it is likely that clinicians were likely to switch sicker patients more rapidly. Secondly, a big part of the study period occurred when the criteria for virologic failure were 10,000 copies/ml and then later 5000 copies/ml [18]. Thirdly, given the absence of third line regimens, clinicians were motivated to preserve second-line ART for inevitable circumstances of need to switch therefore prioritizing switching with sicker patients and delaying switch in healthier patients as long as possible. Lastly, delays in switching could have been due to a reluctance to switch individuals with adherence challenges. Our program has responded to these challenges by retraining clinicians on the need to switch all patients who fail first-line ART as soon as possible after virologic failure, while simultaneously addressing any adherence challenges being faced by patients.

Our rates of switching are not directly comparable to most other studies that report rates of switching to second-line for all patients on ART, rather than for those with confirmed virologic failure. Reported rates of switching in analyses of all patients on ART in various settings range from 2.6 to 4.2/100 pys [9–11]. Using our data to analyze switching rates for our entire ART population (rather than only those with confirmed virologic failure), we found a lower overall rate of switching of 1.2/100 pys, which may be the result of our ability to limit unnecessary switching; whereas, in the absence of VL monitoring, the use of immunologic criteria, which has poor specificity for appropriately identifying virologic failure, results in unnecessary switching [19].

The main limitation to our retrospective analysis was that definition of virologic failure and switching practices may have changed over time as VL monitoring became part of national guidelines as the gold standard to identify treatment failure. It is likely that our definition of virologic failure could have resulted in an under estimate of the rate of switching to second line because previous switching practices could have been more relaxed and therefore not flag some of the patients as virologically failed. We performed a sensitivity analysis using a VL threshold of 5000 copies/ml on two consecutive measurements and found that the rate of switching was slightly higher, but there was no change in the findings from our multivariate analysis outcomes (data not shown). Additionally, we found that the time period (2004–2007 vs. 2008–2011) did not have an effect on the rates of switching irrespective of virologic criteria, implying there were not any time-dependent differences in the switching patterns.

Our study findings can inform the scale-up and optimization of routine VL monitoring in sub-Saharan Africa. In Uganda, the Ministry of Health implemented centralized routine VL monitoring in 2014 and has focused on ensuring access to VL testing for patients on ART, as well as improved turnaround time for returning VL results to the clinics [20]. We recommend that algorithms for the management of confirmed virologic failure and analyses of regimen switch timeliness should be implemented to maximize the benefits of routine VL monitoring for patient outcomes and epidemic control in the 90–90-90 era [21].

## Conclusions

Timely switching of patients failing on first line ART to second line ART regimens can be subjective and delayed. Worsening of immunologic and virologic status of patients may be avoided if switched promptly within first six months of confirmed virologic failure. Implementation strategies that support clinicians to promptly switch patients to second-line ART as soon as virologic failure is confirmed on first-line regimens should be prioritized as routine viral load is scaled up in sub-saharan Africa. Successful interventions will ensure the maximum health benefits are achieved from among patients globally receiving life-saving antiretroviral therapy.

## Additional files

Additional file 1: Table S1. Risk factors of Immunologic decline among HIV infected adults failing on first line ART and switched to second line ART. Cox proportional MSM model of the time to event of immunologic decline defined as decrease in CD4 count ≥50 cells/ul above CD4 count at virologic failure. (DOCX 17 kb)

Additional file 2: Table S2. Risk factors of virologic increase among HIV infected adults failing on first line ART and switched to Second line ART. Cox proportional MSM model of the time to event of virologic increase defined as increase in viral load ≥0.5 log 10 copies/ml above viral load at virologic failure. (DOCX 16 kb)

Additional file 3: Table S3. Risk factors of Immunologic decline, virologic increase or death among HIV infected adults failing on first line ART and switched to Second line ART. Cox proportional MSM model of the time to event of composite endpoint defined as reaching immunologic decline or virologic increase as defined in a and b above, or dying. (DOCX 16 kb)

## Abbreviations

ART: Anti-retroviral therapy
HIV: Human Immunodeficiency Virus
PEPFAR: President’s emergency plan for AIDS relief
RHSP: Rakai Health Sciences Program
VF: Virologic failure
VL: Viral load
WHO: World Health Organization
