# Supplementary material for: Rates of switching to second-line antiretroviral therapy and impact of delayed switching on immunologic, virologic, and mortality outcomes among HIV-infected adults with virologic failure in Rakai, Uganda
Source: BMC Infect Dis. 2017 Aug 22;17:582. doi: 10.1186/s12879-017-2680-6 (PMC5568262; doi:10.1186/s12879-017-2680-6)
Supplement: Supplementary file 2 — Risk factors of virologic increase among HIV infected adults failing on first line ART and switched to Second line ART. Cox proportional MSM model of the time to event of virologic increase defined as increase in viral load ≥0.5 log 10 copies/ml above viral load at virologic failure. (DOCX 16 kb) [file 12879_2017_2680_MOESM2_ESM.docx]

| **Table S2: Risk factors of virologic increase among HIV infected adults failing on first line ART and switched to Second line ART** | | | | | | | |
| --- | --- | --- | --- | --- | --- | --- | --- |
| **Characteristics** | | **n/ pys** | **n /100 pys**  **( 95% CI)** | **Univariate analysis** | | **Multivariate Analysis** | |
|  |  |  |  | **HRs ( 95% CI)** | **p-value** | **HRs ( 95% CI)** | **p-value** |
|  |  |  |  |  |  |  |  |
| **Overall** | | 24/270.1 | 8.9(6.0-13.3) |  |  |  |  |
|  |  |  |  |  |  |  |  |
| **Time to 2nd line ART** | | |  |  |  |  |  |
|  | 0-6 months | 4/107.7 | 3.7(1.4-9.9) | Ref |  | Ref |  |
|  | 7-12 month | 1/75.8 | 1.3(0.2-9.4) | 0.45(0.0-5.3) | 0.520 | 0.32(0.0-3.6) | 0.357 |
|  | 13-24 month | 9/59.1 | 15.2(7.9-29.3) | 10.40(2.0-52.9) | 0.005 | 10.16(1.9-53.0) | 0.006 |
|  | ≥25 months | 10/27.4 | 36.5(19.6-67.8) | 14.24(2.8-72.2) | 0.002 | 10.13(1.7-59.3) | 0.011 |
| **Age in years:** | |  |  |  |  |  |  |
|  | 18-24 | 2/39.0 | 5.1(1.3-20.5) | Ref |  | Ref |  |
|  | 25-34 | 16/150.9 | 10.6(6.5-17.3) | 1.76(0.3-9.8) | 0.514 | 1.87(0.3-12.6) | 0.515 |
|  | ≥35 | 6/80.2 | 7.5(3.4-16.7) | 1.66(0.3-10.4) | 0.583 | 0.96(0.1-7.0) | 0.965 |
| **Gender** | |  |  |  |  |  |  |
|  | Female | 14/173.9 | 8.1(4.8-13.6) | Ref |  | Ref |  |
|  | Male | 10/96.2 | 10.4(5.6-19.3) | 0.56(0.2-1.7) | 0.303 | 0.67(0.2-2.7) | 0.576 |
| **Year of ART Initiation** | |  |  |  |  |  |  |
|  | 2004-2007 | 20/193.3 | 10.3(6.7-16.0) | Ref |  | Ref |  |
|  | 2008-2011 | 4/76.7 | 5.2(2.0-13.9) | 0.61(0.2-2.3) | 0.465 | 1.67(0.5-6.0) | 0.423 |
| **Type of ART treatment Clinic** | |  |  |  |  |  |  |
|  | Central ART clinic | 6/68.2 | 8.8(4.0-19.6) | Ref |  |  |  |
|  | Peripheral ART Clinic | 18/201.8 | 8.9(5.6-14.2) | 0.70(0.2-2.7) | 0.596 |  |  |
| **WHO Stage at ART Initiation** | |  |  |  |  |  |  |
|  | 1 | 7/75.2 | 9.3(4.4-19.5) | Ref |  |  |  |
|  | 2 | 8/111.6 | 7.2(3.6-14.3) | 0.66(0.2-2.4) | 0.525 |  |  |
|  | 3 or 4 | 9/83.3 | 10.8(5.6-20.8) | 1.42(0.4-5.4) | 0.601 |  |  |
| **First Line ART regimen** | |  |  |  |  |  |  |
|  | EFV based regimen | 8/71.2 | 11.2(5.6-22.5) | Ref |  |  |  |
|  | NVP based regimen | 16/198.8 | 8.0(4.9-13.1) | 1.69(0.5-5.5) | 0.381 |  |  |
| **CD4 count at ART initiation (cells/ul)** | | |  |  |  |  |  |
|  | ≥100 | 17/144.0 | 11.8(7.3-19.0) | Ref |  | Ref |  |
|  | ≤99 | 7/126.1 | 5.6(2.6-11.6) | 0.28(0.1-0.9) | 0.033 | 0.56(0.1-2.2) | 0.408 |
| **Time from ART start to virologic failure** | | | |  |  |  |  |
|  | 0-24 months | 20/234.0 | 8.5(5.5-13.2) | Ref |  |  |  |
|  | ≥25 months | 4/36.0 | 11.1(4.2-29.6) | 1.33(0.3-6.5) | 0.726 |  |  |
| **Year of virologic failure** | |  |  |  |  |  |  |
|  | 2005-2007 | 12/130.3 | 9.2(5.2-16.2) | Ref |  |  |  |
|  | 2008-2013 | 12/139.8 | 8.6(4.9-15.1) | 1.09(0.4-3.2) | 0.878 |  |  |
| **Virologic suppression prior to virologic failure** | | | |  |  |  |  |
|  | No | 15/141.4 | 10.6(6.4-17.6) | Ref |  |  |  |
|  | Yes | 9/128.6 | 7.0(3.6-13.4) | 0.99(0.3-3.1) | 0.983 |  |  |
| EFV=Efavirenz, NVP=Nevirapine; n= Number switched to 2nd line; pys: Person years of observation; virologic failure = Incident first line ART failure; HRs= Hazard Ratios | | | | | | | |
